# Recommendations for the Development of Socioeconomically-Situated and Clinically-Relevant Neuroimaging Models of Pain

**DOI:** 10.3389/fneur.2021.700833

**Published:** 2021-09-07

**Authors:** Marianne C. Reddan

**Affiliations:** Department of Psychology, Stanford University, Stanford, CA, United States

**Keywords:** chronic pain, neuroimaging biomarkers, translational ability, social epidemiology, social determinants of health, machine learning, biopsychosocial pain models

## Abstract

Pain is a complex, multidimensional experience that emerges from interactions among sensory, affective, and cognitive processes in the brain. Neuroimaging allows us to identify these component processes and model how they combine to instantiate the pain experience. However, the clinical impact of pain neuroimaging models has been limited by inadequate population sampling – young healthy college students are not representative of chronic pain patients. The biopsychosocial approach to pain management situates a person's pain within the diverse socioeconomic environments they live in. To increase the clinical relevance of pain neuroimaging models, a three-fold biopsychosocial approach to neuroimaging biomarker development is recommended. The first level calls for the development of diagnostic biomarkers *via* the standard population-based (nomothetic) approach with an emphasis on diverse sampling. The second level calls for the development of treatment-relevant models *via* a constrained person-based (idiographic) approach tailored to unique individuals. The third level calls for the development of prevention-relevant models *via* a novel society-based (social epidemiologic) approach that combines survey and neuroimaging data to predict chronic pain risk based on one's socioeconomic conditions. The recommendations in this article address how we can leverage pain's complexity in service of the patient and society by modeling not just individuals and populations, but also the socioeconomic structures that shape any individual's expectations of threat, safety, and resource availability.

## Introduction

Neuroimaging models have significantly expanded our understanding of the neural processes that instantiate a person's subjective pain experience [for reviews see (1–3)]. Through neuroimaging, we have learned that the brain representation of pain is highly distributed and multidimensional involving sensory, cognitive, and affective components (4–7). Neuroimaging models employing multivariate [i.e., multivoxel pattern analysis or MVPA; (8)], predictive (i.e., machine learning), and network analysis techniques can, respectively, delineate multiple component processes that contribute to both acute and chronic pain (7, 9–11), predict a person's self-reported evoked pain intensity (12, 13), and localize sites of functional connectivity disruption across chronic pain phenotypes (14, 15).

Despite these important advances, neuroimaging research has yet to significantly impact the clinic. Anatomical and resting state markers lack specificity- it remains unknown whether changes are due to chronic pain or to co-morbidities like anxiety and depression [for reviews see (16, 17)]. Furthermore, most models are developed on experimental data of evoked phasic pain where participants experience a brief (under 12 s) noxious stimulus such as prick or a hot plate against the skin. This does not translate well to chronic pain which must persist 3 or more months. Acute or phasic pain is typically appraised as temporary and separate from the self, while chronic pain is typically appraised as unending and apart of one's life (18). Chronic pain is also highly personalized and embedded in spontaneous and tonic, rather than evoked and phasic, activity in the brain (19–22). Finally, population samples are not well-stratified across economic class, race, or ethnicity (23). In most cases, participant socioeconomic status (SES) is not reported nor well-measured [for a review see (24)]. Because chronic pain disproportionately affects the poor and working class across the globe (25–33), neuroimaging models of pain must take socioeconomic information into account.

The biopsychosocial approach to pain management attempts to encapsulate the broader societal issues which situate interactions among the biological, psychological, and social components of the pain experience (34). This conceptual framework states that understanding pain requires an understanding of the whole patient, their relationships, and society (35, 36). However, the biopsychosocial approach is largely theoretical and has yet to be well-integrated into pain neuroimaging research. To resolve this translational gap, this perspective formulizes the biopsychosocial approach into testable neuroimaging models intended for the diagnosis, treatment, and prevention of chronic pain. These models endeavor to predict and understand chronic pain from three levels, that of the individual, of the population, and of society.

First, recommendations are made to increase the diagnostic relevance of the population-based, or nomothetic, approach to the development of pain neuroimaging models. These recommendations include a shift in focus from evoked phasic pain to evoked tonic pain paradigms and the recruitment of larger and more diverse population samples. Second, a person-based or idiographic approach to the development of treatment-relevant models is discussed. Recommendations are made for the training and implementation of these models so that they can be used to track disease progress and treatment efficacy within individual patients. Finally, a novel society-based, or social epidemiological approach to the development of prevention-relevant models is proposed. This approach situates an individual's disease state within the socioeconomic conditions they live in. Lastly, implications for both the clinic and public policy are outlined.

## Nomothetic (Population-Based) Approach to Diagnostic Models

**Figure 1:**
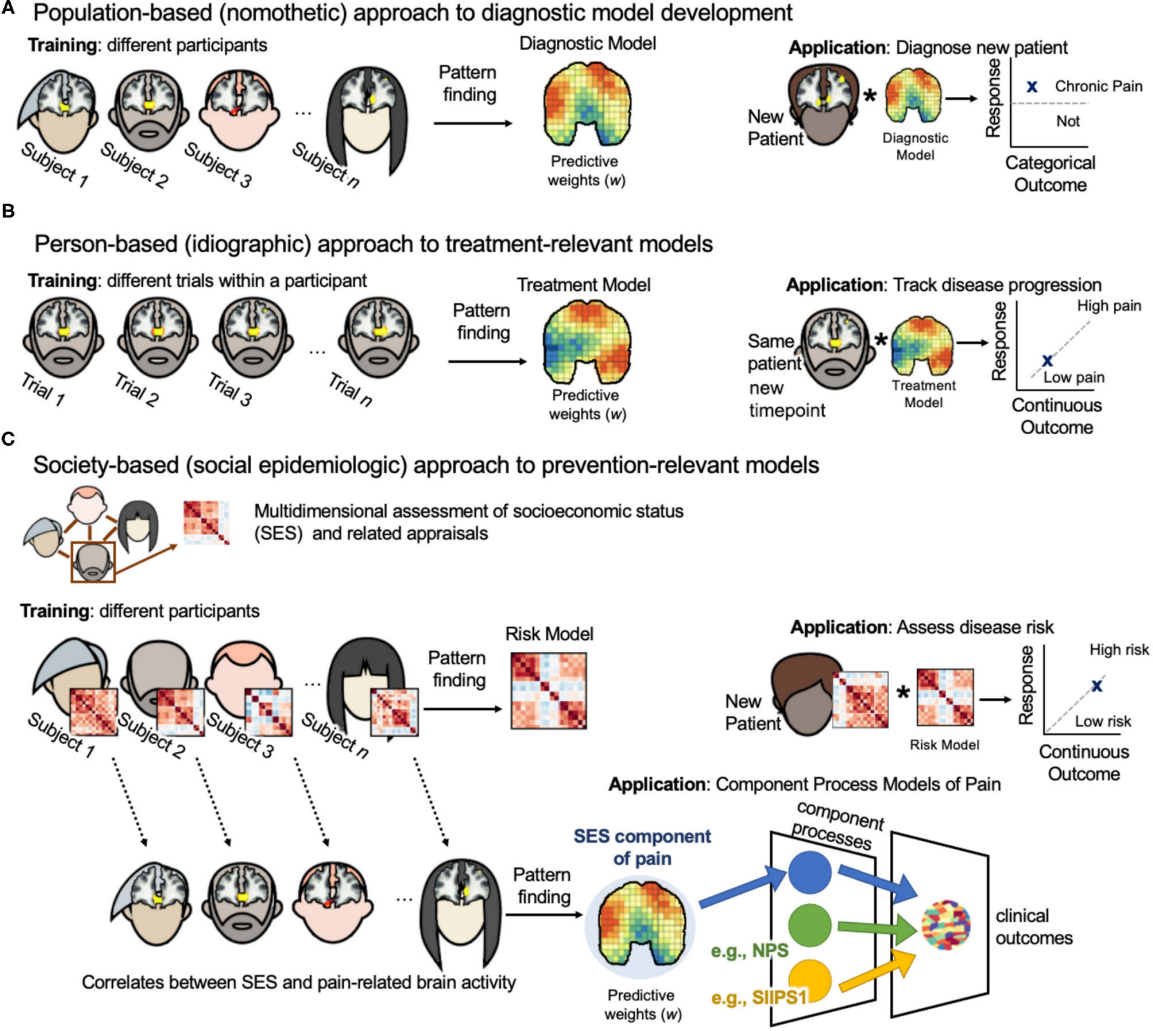
Three-level biopsychosocial approach to neuroimaging biomarker development. **(A)** Population-based (nomothetic) approach to diagnostic model development. Neuroimaging model weights are estimates of population-level associations between brain activity and pain outcomes (i.e., diagnostic category vs. healthy control). Population samples should be large and diversely sampled across gender identity, race, and socioeconomic identities. Models should be validated on external clinical data sets. Models can then be applied to the brain activity of a new patient to diagnosis their pain condition. **(B)** Person-based (idiographic) approach to treatment-relevant models. Neuroimaging model weights are estimates of person-level associations between brain activity and pain outcomes (i.e., pain severity) for the same person through time. Models weights can be regulated by nomothetic models to lessen demands on data collection from one patient. Models can be applied in the same patient at later time points to assess their disease progression or to assess treatment efficacy. Such models can be used to tailor treatment selection on a case-by-case basis. **(C)** Society-based (social epidemiologic) approach to prevention-relevant models. This approach requires two steps. First, participants complete a multidimensional survey that assesses both their environment (i.e., socioeconomic status) and their personal internalization of these conditions (Table 1). Then, a risk model is trained on these survey data to predict pain severity. The weights of this risk model are estimates of population-level associations between a person's socioeconomic conditions and pain outcomes. This model can be applied to the survey data of a new patient to assess their risk of pain chronification. Person-level survey data can be related to person-level pain-related brain activity, and then a neuroimaging model of the SES component of pain processing can be developed. Neuroimaging model weights are estimates of group-level associations between the socioeconomic conditions a person lives in and their pain-related brain activity. Such a model could be combined with other neuroimaging component process models of pain, such as the NPS and SIIPS1, to predict clinical outcomes in new patients.

Human subjects research is largely nomothetic, that is, the goal is to generate an explanation of brain activity that is “universal” and generalizable to entire populations (Figure 1A). Such models are trained on many different people sampled from the same population. Individual differences are treated as noise and intentionally minimized through careful inclusion/exclusion criteria, outlier removal, and the inclusion of confound regressors controlling for demographic variables such as age and gender identity [for a review see (37)]. The nomothetic approach is appropriate for the development of diagnostic biomarkers because inferences must be drawn from the wider population to identify pain pathologies in new patients presenting symptoms for the first time.

Nomothetic neuroimaging model weights are estimates of population-level associations between brain activity and pain outcomes (i.e., self-reported pain intensity). Models are cross-validated via an iterative “leave-*N*-subjects-out” procedure to assess performance on out-of-sample participants [for recommendations see (38)]. Next, they are validated on held out “validation sets”; though this external validation process is not common in single neuroimaging studies due to the demand on sample size. More often, this validation process occurs over a series of papers across unique data sets collected on different scanners in varied locations [for a review see (2)]. This a slower validation process, but it is a more thorough and robust one. Once validated, the model's predictions are deemed suitable for application to a new individual drawn from the same population.

A strength of this approach is its ability to identify separable component processes of pain (7). For example, the neurologic pain signature (NPS) is a well-validated model for acute pain evoked by noxious events (13). It captures a component process that contributes to the perceived intensity of an acute painful stimulus. It includes patterns of activity in the anterior cingulate, somatosensory cortex, and periaqueductal gray. Woo et al. (7) developed a separate multivariate predictive model of pain called the stimulus intensity independent pain signature-1 (SIIPS1). SIIPS1 captures fluctuations in pain independent of noxious stimulus intensity. It includes activity in the nucleus accumbens, lateral prefrontal cortex (PFC), and parahippocampal cortex. When combined with the NPS, the two explain more variance in brain activity than either model alone. However, the combined variance explained is 30%, indicating that there are more component processes relevant to evoked pain experiencing that have yet to be discovered (Figure 1C).

Though the NPS and SIIPS1 can predict different aspects of acute pain experiencing, they cannot distinguish between chronic pain patients and controls. It is unclear whether models trained on evoked phasic pain are informative for the diagnosis of chronic pain. To distinguish between fibromyalgia patients and healthy controls, the NPS was subdivided into its positive activations and then combined with a multisensory model similar to SIIPS1 and a separate model trained to predict evoked pain in fibromyalgia patients (9). The combinatorial model performed with high accuracy within the study it was developed, however, it is unknown how it performs in external data sets. Combining models like this may be prone to overfitting, so the preregistration of model combinations is recommended.

The translational limitations of evoked phasic pain models may be due to the phasic, rather than the evoked, nature of the noxious stimuli. Recently, a tonic pain neuroimaging biomarker with clinical relevance was developed. This biomarker, called TOPS, was trained on evoked *tonic* pain trails in healthy controls (39). In this experiment, capsaicin was placed on the tongue to evoke pain for 1-2 min. TOPS can predict clinical pain severity and distinguish between patients and controls in two independent studies of chronic low back pain. It is possible that tonic stimulations hold greater clinical utility than phasic because longer stimulations allow for rumination and the activation of resting state networks that may play a role in the chronification of pain (22, 40, 41).

TOPS was able to track within-individual variations in pain avoidance ratings with an average correlation of *r* = 0.51. Though this holds promise for the clinic, there is still much variance left to be explained. Pain is an idiosyncratic experience with many dimensions; therefore, the nomothetic approach may never be able to explain the entirely of an individual's pain experience, however, a “good enough” approximation might be achieved through the development of a suite of component process models that can be combined on a person-by-person basis. As we build more models of pain components, such as social context, interoception, affect, and expectations for pain relief, we may begin to chip away at this complex neural representation.

To this end, I make the following recommendations: First, a concerted effort must be made to recruit larger, more representative samples of the population. Nomothetic models are only suitable for application on new individuals drawn from the same population in which they were trained. The NPS was trained on only 20 participants, eight of which are women and 79% are White. Sampling procedures which primarily recruit from the student pool of the universities where the research is conducted unintentionally select for young high income and high education level White participants not of Hispanic origin (23). This is not representative of the world at large, nor is it representative of populations suffering from chronic pain. In the United States, most chronic pain patients are low-education and low-income women of color over the age of 45 (26, 42, 43).

Funding agencies must provide sufficient support so that researchers can expand their recruitment, possibly by employing companies that specialize in representative sampling to stratify samples across age, gender identity, race, ethnicity, wealth and income, education level, and personality traits. Second, pain models and pain data sets should be made open and shareable to increase collective clinical impact. Patient data sets, especially those involving spontaneous pain paradigms, are difficult to collect, but are the most clinically-relevant. With increased data sharing, new pain components developed in easier to collect (i.e., evoked pain in healthy controls) diverse populations can be validated in clinically-relevant samples to improve translation and impact.

## Idiographic (Person-Based) Approach to Treatment-Relevant Models

Pain is heterogeneous. The nomothetic assumption that “one-size-fits-all” ignores diversity in economic class, cultural background, gender identity, ethnicity, and personality, and limits applicability in real-world pain treatment. For example, emotional pain is positively correlated with physical pain at the group level, but this relationship is inconsistent across time within unique individuals (44). Indeed, neither SIIPS1 nor TOPS positively predicts pain in each individual the model was trained on; approximately 2-3% of the training data show effects in the opposite direction. It is possible that one's unique experiences with pain can influence the magnitude or direction of the relationship a pain component process has on their individual pain response. The idiographic approach accounts for variance across individuals by allowing for *personalized* predictions. Individual differences in pain expression have made it difficult for biomarkers to be developed on lower dimensional data like facial expressions, skin conductance responses, and heart rate, however, recent idiographic approaches to modeling these types of data have significantly improved their predictive power (45–47). In the clinic, such models may provide objective assessments of disease progression and treatment progress.

In the person-based approach, models are trained on many different samples from the same individual (Figure 1B). This commonly involves estimating pain-related brain activity from single trials within one experimental session. Predictive brain maps developed on one participant should be internally cross-validated to test the model's ability to predict pain outcomes on out-of-sample trials from the same participant. While it might be useful to validate the model on later timepoints, current evidence suggests that there is stability in a single individual's network-level representation of the same stimulus through time (48).

Advantages of these models include improved accuracy and the ability to capture representations at finer spatial scales [e.g., (49–53)]. Because idiographic models require hours of data acquisition from a single participant, it can be difficult to collect from patients. One way to reduce the demands on scan time is to constrain the idiographic model with nomothetic priors. For example, Lindquist et al. (52) regularized an idiographic model of acute pain in healthy controls with the NPS. The regularized model performed better than both the NPS and a purely idiographic model trained on that subject's data alone. This method of regularization is known as group-regularized individual prediction (GRIP). It combines population-based and idiographic models in proportion to their variances. It does this by applying a shrinkage factor to the model weights. The shrinkage factor penalizes idiographic activity that appears unlikely (i.e., noise) relative to group activity.

Non-regularized idiographic models are still likely to be useful if sufficient data are collected from the patient. The recommendation here is to compare the performance of regularized and non-regularized idiographic models within patients and select the best model on a patient-by-patient basis. This patient-tailored model can later be applied to their own brain activity in longitudinal follow-ups and intervention paradigms to track disease progress and treatment efficacy. It could also be deployed in real-time neurofeedback paradigms where participants can test multiple interventions and empirically validate which works best for them [see (54)]. Within this framework, a diversity of treatments (e.g., drugs, expectancy manipulations, placebo interventions, self-regulation, or mindfulness) can be tested with reduced bias.

## Social Epidemiologic (Society-Based) Approach to Prevention-Relevant Models

Studies of global chronic pain prevalence suggest that societal stressors may contribute to the chronification of pain (32, 55–58). This is not surprising–the relationship between one's economic class and chronic illness has been observed as early as 1848, when Rudolph Virchow determined that treating the Typhus epidemic in Upper Silesia would require more than medicine. Virchow prescribed changes to the material conditions of the people whom the epidemic most severely impacted—the poor and working class (59). He concluded that though all illness has a biological origin, where it spreads and who is most susceptible is determined by structural factors such as housing, working conditions, diet, and sanitation (60). Similar observations have been made about chronic pain today. When controlling for age, race, and education level, a study conducted in an urban trauma center found that homelessness and low income were strongly associated with chronic pain (27).

Relationships between low economic class and chronic pain prevalence have been found across the United States (26, 61, 62) as well as across different cultures and countries including South Africa (63), Brazil (31), Iran (64), Germany (65), Austria (56), Sweden (66), Finland (67), the United Kingdom (25, 68), Japan (28), Nepal (33), and South Korea (69). Despite the long history and geographic spread of these associations, SES has largely been ignored by pain neuroimaging research. There are several reasons for this: First, there is little communication between epidemiologists and neuroimagers [an effort to correct this has begun, see (70)]. Second, the lack of socioeconomic diversity in research samples obfuscates this connection. Finally, it is difficult to mathematically relate complex social structures to functional brain activity. To the author's knowledge, only one neuroimaging study has done this to date (10). Here I propose to resolve this gap with a social epidemiologic approach to neuroimaging models of chronic pain.

Social epidemiologists study how socioeconomic structures, institutions (i.e., law, education), and social relationships influence health outcomes. A social epidemiologic approach to neuroimaging models of pain relates the structure of society to brain health and function. The primary goal of this approach is chronic pain prevention. The first step is to collect survey data assessing an individual's socioeconomic conditions and subjective experience of social status. This multidimensional assay can then be applied to pain-related brain activity to develop a neuroimaging model of socioeconomic contributions to chronic pain (Figure 1C). The resulting SES neuroimaging model may be a component process of pain useful for combinatorial models described earlier. This approach may allow us to identify patients most at risk for pain chronification because one's internalization of their socioeconomic conditions may play a role in the onset and maintenance of chronic pain (58, 61, 71).

The transition from acute to chronic pain is marked by a shift in processing from nociceptive components to socioemotional components of pain—specifically, PFC-limbic circuitry, including the NAc/striatum, amygdala, and hippocampus (72, 73), and the default mode network [DMN; (41)]. Changes to PFC-limbic circuitry may indicate a change in the valuation of pain (11, 74). Changes to DMN connectivity may change how the pain experience is construed in relation to the self (75, 76). Both of these networks are altered by poverty and socioeconomic stress (77). Activity in the PFC (78, 79) and ventral striatum (80) differs as a function of SES during both valuation and the processing of self-related information (81–83). Childhood poverty is correlated with aberrant functional connectivity within the DMN (84, 85). Interestingly, these aberrations can be reversed in people who have high income later in life (86). Relatedly, (10) found a threshold in annual income (>$25,000) that delineated vulnerability from protection in chronic pain patients. In the United States, the poverty line for a family of four is $26,200; meaning families that make less than this cannot afford food, rent, and other basic needs (87). It is unknown whether changes in income can reverse chronic pain status, however, chronic pain patients of high SES tend to have better clinical outcomes (88).

The impact of socioeconomic stress on chronic pain may not be reducible to income alone. The experience of social strain or subordination itself may contribute to chronic illness above and beyond income-level (89, 90). In non-human primates low social status is associated with immune system deficits that increase risk of infection and slow wound healing (91, 92). Chronic social stress may underlie immunosuppression in humans and animals [for a review see (93)]. People in lower social classes have a lower sense of personal control which is associated with higher levels of stress and pain (94). However, a high sense of self-efficacy is protective against chronic pain and pain severity (95). The protective effect of self-efficacy may be independent of class. For example, a large study in South Korea (*N* = 28,532) demonstrated that when controlling for monthly income, the presence of labor unions reduced low back pain prevalence (69). Another study in the United States found that unionized workers experience less severe pain for work-related musculoskeletal disorders (96). One interpretation of these effects is that labor unions change perceptions of self-efficacy, pain controllability, and expectations for care and safety by giving worker's the ability to advocate for themselves through collective bargaining (97).

A major barrier to the study of socioeconomic factors in chronic pain is the lack of a standardized assessment of SES. Here I propose the creation of a “Pain-Predispositions Profile Survey” (Table 1), a multidimensional assay of debt, income, property ownership, investments/savings, family wealth, education, perceived social status, environment (urban or rural), housing situation, childhood attachment, SES-related personality/evaluative traits (i.e., pain catastrophizing, controllability perceptions), as well as measures of income inequality within the city and country the patient resides in. A predisposition model of chronic pain can then be developed on these survey data that predicts patient pain status or severity. A cross-validated procedure similar to that employed by Vachon-Presseau et al. (10) can then be used to relate the survey data to functional networks in chronic pain patients (or healthy participants in evoked pain paradigms) to uncover a socioeconomic-related component process contributing to the pain experience (Figure 1C). Neuroimaging may not always be an available tool for the diagnosis and treatment of chronic pain—the survey-based model, however, is scalable and can be leveraged for treatment selection by matching people on survey similarity. Treatment programs that are validated on patients in neuroimaging studies can then be recommended to new patients with greater confidence.

**Table 1:** Socioeconomic Pain-Predispositions Profile Survey.

**Level of assessment**	**Profile dimension**	**Examples**
External conditions	Objective SES	Annual income, debt, amount of money in savings or investments, property ownership, housing status, number of people in a household, marital status, education level, parental education level, employment status, type of occupation, health insurance status, union membership
	Demographic Information	Age, race, gender identity, location of residence (urban vs. rural), location of birth, ethnicity, immigration status
	Sociopolitical Environment	Type of government in the country of residence, the gross domestic product (GDP) of country of residence, level of income inequality in country and city of residence, type of economic system in country of residence
Internalization of external conditions	Perceived SES	“I have a very high standing in my workplace or community.” “If I got sick I would be able to access quality care.” from The MacArthur Network on SES and Health and The MacArthur Scale of Subjective Social Status (98, 99)
	Perceived Social Support	“There is a person in my life who is around when I am in need.” from the Multidimensional Scale of Perceived Social Support (100)
	Job satisfaction	“Over the past 12 months, have you ever experienced workplace discrimination based on your race, gender, education, etc?” from Perceived Workplace Discrimination (69) “I can get positive feed-back and respect in my work.” from Work Satisfaction Index (101)
Beliefs about external conditions	System Justification	“Society is set up so that people usually get what they deserve.” from General and Economic System Justification (102)
	Social Trust	“I feel that people generally earn the rewards and punishments that they get in this world.” from Just World Scale (103) see also ‘Kind of Person’ Implicit Theory Scale (104)
	Perceptions of Self-efficacy	“Relief from pain is chiefly controlled by doctors” from Beliefs about Controlling Pain (105) see also, Locus of Control Scale (106)
Personality	Personality Type	“I am moody, tense, and lack self-confidence.” from the Big 5 Personality Inventory (107)
	Emotional reactivity	“I often have concerned feelings for people less fortunate than me.” “I sometimes feel helpless when I am in the middle of a very emotional situation.” from the Interpersonal Reactivity Index (108)
	Attachment Style	“I find it difficult to allow myself to depend on others.” from the Attachment Style Questionnaire (109)
	Pain catastrophizing	“When I am in pain I feel I can't go on.” “I keep thinking of other painful events” from The Pain Catastrophizing Scale (110)
	Trait anxiety and depression	“I worry too much over something that really doesn't matter” from the State Trait Anxiety Inventory (111) see also Beck Depression Inventory (112)

*A multidimensional assay of socioeconomic conditions, their internalization, and pain-related appraisals and personality traits*.

## Discussion

An individual's valuation of a painful event (113–115), their expectations for support and health care (116–118), their beliefs about pain permanence (119, 120), personality traits (10, 121), and the socioeconomic conditions they exist in (10, 122) influence their brains' representation of pain. Pain, therefore, is a personal experience instantiated by biological processes and situated within one's socioeconomic conditions. Neuroimaging models situated within the socioeconomic structures of the population being studied are necessary for the development of a more complete understanding of the complexities of human pain. In this perspective, I discuss how three approaches to the development of pain neuroimaging models—nomothetic (population-based), idiographic (person-based), and social epidemiologic (society-based)—can be applied to the diagnosis, treatment, and prevention of chronic pain. These three approaches taken together serve to operationalize the biopsychosocial model of pain within a neuroimaging context.

It is estimated that 1% of the world's population controlled 44.8% of the world's wealth in 2018 (123). Economists from varied and opposing points on the political spectrum agree that an increasingly globalized and automated economy will heighten existing barriers to economic mobility and make income inequality more stark, widespread, and permanent (124). Therefore, it is my final recommendation that scientists and clinicians advocate for chronic pain patients at the level of public policy. In the words of Virchow, “Disease is only a manifestation of life under pathological conditions… Medicine is a social science and politics is nothing else but medicine on a large scale.”

## Data Availability Statement

The original contributions presented in the study are included in the article/supplementary material, further inquiries can be directed to the corresponding author/s.

## Author Contributions

The author confirms being the sole contributor of this work and has approved it for publication.

## Funding

NIMH (R01 MH112560), Computational and brain predictors of emotion cue integration (PI: Zaki).

## Conflict of Interest

The author declares that the research was conducted in the absence of any commercial or financial relationships that could be construed as a potential conflict of interest.

## Publisher's Note

All claims expressed in this article are solely those of the authors and do not necessarily represent those of their affiliated organizations, or those of the publisher, the editors and the reviewers. Any product that may be evaluated in this article, or claim that may be made by its manufacturer, is not guaranteed or endorsed by the publisher.
